# Biological functions and applications of LncRNAs in the regulation of the extracellular matrix in osteoarthritis

**DOI:** 10.3389/fcell.2023.1330624

**Published:** 2024-01-08

**Authors:** Di Shi, Yufeng Mei, Wan Hao, Jun Li, Shuguang Liu, Xiao Lin

**Affiliations:** ^1^ Laboratory for Bone Metabolism, Xi’an Key Laboratory of Special Medicine and Health Engineering, Key Laboratory for Space Biosciences and Biotechnology, Research Center for Special Medicine and Health Systems Engineering, NPU-UAB Joint Laboratory for Bone Metabolism, School of Life Sciences, Northwestern Polytechnical University, Xi’an, Shaanxi, China; ^2^ Department of Joint Surgery, Honghui Hospital, Xi’an Jiaotong University, Xi’an, Shaanxi, China; ^3^ Research and Development Institute of Northwestern Polytechnical University in Shenzhen, Shenzhen, China

**Keywords:** osteoarthritis, lncRNAs, extracellular matrix, chondroctyes, gene therapy

## Abstract

Osteoarthritis (OA) is a major cause of disability, characterized by chronic pain, irreversible destruction, and loss of function of the articular cartilage. The integrity and arrangement of the composition and structure of the extracellular matrix (ECM) are essential for maintaining the elasticity, integrity, and mechanical support function of the cartilage tissue. Osteoarthritis causes substantial changes in the ECM, driving the progression of the disease. Recent studies have shown that the ECM plays a critical role in the development of cartilage tissue as well as the occurrence and development of osteoarthritis by directly or indirectly regulating chondrocyte proliferation, apoptosis, differentiation, and gene expression. Long non-coding RNAs (lncRNAs) are a class of non-coding RNAs derived from large transcripts. Mutations and disorders of lncRNAs are closely related to the development of osteoarthritis. Abnormal expression of lncRNAs in osteoarthritic cartilage regulates the synthesis and decomposition of the cartilaginous ECM. Therefore, the use of lncRNAs as nucleic acid drugs that regulate their targets may reduce ECM degradation, thereby delaying the pathological progression of osteoarthritis. In this review, the regulatory effects of lncRNAs on ECM in different cell behaviors related to OA are summarized. The roles of lncRNAs in the proliferation, apoptosis, differentiation, and ECM-related gene activity of chondrocytes, as well as the application of lncRNAs as potential gene therapy drugs for the repair and regeneration of osteoarthritic tissue, are also reviewed. A better understanding of the roles of lncRNAs in guiding chondrocyte behavior and ECM metabolism is critical for their future applications in osteoarthritis therapy and regenerative medicine.

## 1 Introduction

Osteoarthritis (OA) is a common chronic inflammatory disease that may occur in any joint throughout the body and is a painful condition resulting in the irreversible destruction of articular cartilage, leading to pain, disability, and loss of function (Hunter and Bierma-Zeinstra, 2019; Cao et al., 2021). Among individuals aged 70 years and older, OA ranked as the seventh leading cause of years lived with disability, and the aging global population is contributing significantly to a substantial rise in the number of new cases of OA (Quicke et al., 2022). The high cost of treatment increases the economic burden of OA, particularly in low- and middle-income countries (Steinmetz et al., 2023). Therefore, the prevention and treatment of OA is an urgent problem to be solved.

The extracellular matrix (ECM) is a three-dimensional polymeric structure that is a vital component of all tissues, crucial for sustaining life (Theocharis et al., 2016). The roles of ECM go beyond its physical support for tissue elasticity and integrity, and it is a dynamic structure that regulates tissue homeostasis through continuous remodeling (Hynes, 2009). The integrity of ECM composition and structure is essential to the normal function of tissues such as cartilage, liver, and muscle. For example, decompensated hepatic fibrosis leads to cirrhosis, and the accumulation of fibrotic ECM impedes the liver’s ability to effectively exchange fluids (Neshat et al., 2021). The structure and arrangement of the ECM play a crucial role in the normal functioning of bone and cartilage tissue. Diseases such as osteoarthritis cause substantial alterations of the ECM, which not only drive the progression of the disease but also contribute to the development of its primary symptoms (Krishnan and Grodzinsky, 2018). Recent studies have shown that the ECM directly or indirectly influences almost all cellular behaviors, including proliferation, apoptosis, differentiation, and gene expression, making it indispensable in the development of normal cartilage, as well as the occurrence and development of OA (Peng et al., 2021).

Long noncoding RNAs (lncRNAs) are a class of noncoding RNAs derived from larger transcripts (>200 nt in size) that are not translated into proteins (Winkle et al., 2021). In recent years, there has been growing interest in the study of lncRNAs, as they are thought to play regulatory roles in diverse physiological and pathological processes. Recent findings suggest that lncRNAs play crucial roles in cell differentiation, lineage specification, organ development, and tissue homeostasis (Schmitz et al., 2016). Mutations and dysregulation of lncRNAs are closely associated with the development of various complex human diseases. Furthermore, lncRNAs play an important role in the development of bone and cartilage. Previous studies have shown that lncRNA DANCR inhibits the Wnt/β-catenin signaling pathway through CTNNB1, thereby regulating the osteogenic differentiation process in osteoporosis (Wang et al., 2020a). Ectopic expression of lncRNA AC008 significantly decreased the viability of chondrocytes, while also promoting apoptosis and ECM degradation of arthritis (Yang et al., 2021). LncRNA AC006064.4–201 directly interacts with PTBP1 to destabilize CDKN1B mRNA and reduce CDKN1B translation, whereby the AC006064.4-201/PTBP1/CDKN1B axis plays an important role in the occurrence and development of OA (Shen et al., 2023). Abnormal expression of LncRNAs in OA cartilage regulates the synthesis and breakdown of the cartilaginous ECM. Therefore, using LncRNAs as small nucleic acid drugs regulating their targets may alleviate ECM degradation, inflammation-mediated chondrocyte apoptosis, chondrocyte differentiation, and related processes, thus delaying the pathological progression of OA (Pearson and Jones, 2016).

This review summarizes the most important regulatory events involving lncRNAs in the development of OA from a clinical, cellular, and *in vivo* perspective, focusing on the major mechanisms through which lncRNAs influence the ECM of cartilage in the process of regulating the proliferation, differentiation, and apoptosis of chondrocytes. At the same time, we summarize how lncRNAs can be used as biomarkers for early diagnosis of OA as well as to provide novel nucleic acid drugs for clinical treatment.

## 2 Changes in the extracellular matrix related to the pathogenesis of osteoarthritis

The main component of articular cartilage is hyaline, which is a clear, smooth, lubricated surface with a thickness of approximately 2 mm in most human joints (Shepherd and Seedhom, 1999; Hunziker et al., 2002). Bone joints are composed of a large number of rich ECM components, which include collagen, hyaluronic acid, glycoproteins, proteoglycans, and chondrocytes (Rim et al., 2020), the latter being the only cells in articular cartilage. Severe mechanical stimulation, genetic predisposition, obesity, age, estrogen deficiency, and other factors may contribute to the degeneration of articular cartilage, which leads to the occurrence and development of OA (Zhang et al., 2022a). During the progression of arthritis, the structure of the joint changes, mainly manifesting as cartilage degradation, osteophyte formation, joint space narrowing, synovial inflammation, and loss of normal joint function, which can eventually lead to disability (Martel-Pelletier et al., 2016).

Studies have shown that destructive changes in OA patients leading to the loss of articular cartilage are related to abnormal functioning of chondrocytes, including chondrocyte proliferation, apoptosis, differentiation, and accompanied ECM degradation (Fujii et al., 2022). These changes are reflected in a molecular disorder mainly presenting as abnormally decreased anabolism of type II collagen and aggrecan, along with an abnormal increase of catabolism caused by degradative enzymes such as metalloproteinases (MMPs) and aggrecan-degrading enzymes (a disintegrin and metalloproteinase with thrombospondin-like motifs, ADAMTS) in joint tissue (Troeberg and Nagase, 2012; Tang et al., 2022a). Fibrillar type II collagen is one of the main ECM components of articular cartilage. Its stability and strength provide articular cartilage with integrity and resiliency to stress (Theocharis et al., 2016). In OA, MMPs (primarily collagenase and gelatinase) are involved in the degradation of hyaluronan and linking proteins to provide cartilage with resistance to compression and the ability to absorb shocks (Kiani et al., 2002). The anabolic process of chondrocyte aggrecan recombination is very dynamic during the development of OA. The domains of aggrecan protein contain several cutting sites susceptible to MMPs, ADAMTS, and other enzymes (Santamaria and Yamamoto, 2020). The loss of aggrecan renders the collagenous framework susceptible to both mechanical attrition and enzymatic attack (Ismail et al., 2016; Miller et al., 2018), leading to the cleavage and denaturation of collagen fibrils in the tissue (Powell et al., 2019). In particular, MMP-1, -8, and -13 can cut the site between glycine 775 and leucine 776 in the triple helix structure of collagen, resulting in helix unwinding, which in turn produces two new shorter peptides. The levels of these two shorter peptides were found to be increased in patients with OA compared to healthy controls (Bay-Jensen et al., 2022). Aggrecan is another core structural protein in the cartilage ECM that presents as the initial histological indication of cartilage deterioration in arthritis and fully demonstrates the importance of aggrecan degradation in cartilage tissue for OA (Bay-Jensen et al., 2022). ADAMTS proteins are a superfamily comprising 26 secreted zinc-dependent endopeptidases. Most ADAMTS proteins can break down ECM components and participate in normal embryological or physiological processes. In OA, ADAMTS4 and ADAMTS5 contribute to the cleavage of aggregates, whereby the latter plays a dominant role. Notably, ADAMTS5-deficient mice are resistant to surgically induced OA (Mead and Apte, 2018). Therefore, type II collagen and aggrecan are necessary for cartilage formation, while MMPs and ADAMTS are adverse factors in OA.

## 3 Biosynthesis and function of lncRNAs

LncRNAs are traditionally defined as transcripts with 200 or more nucleotides that do not possess the potential for encoding proteins, derived from intergenic or overlapping protein-coding regions of genes (Ali et al., 2021). LncRNAs are synthesized in the nucleus, and most are transcribed from intergenic, exonic, or distal protein-coding regions of the genome by RNA polymerase II. To improve their stability, immature lncRNAs are subjected to post-transcriptional modification, including polyadenylation at the 3′ end and capping at the 5’ end. Subsequently, 98% of lncRNAs undergo selective splicing (Statello et al., 2021). LncRNAs contain a smaller number of exons and thus produce shorter transcripts after splicing. The final step in lncRNA biogenesis is the formation of thermodynamically stable structures that allow them to interact with proteins, DNA, and other RNA molecules (Dahariya et al., 2019).

Mature lncRNAs can be found in the nucleus or cytoplasm. LncRNAs in the nucleus control the expression of genes by interacting with DNA, chromatin-modifying complexes, and a variety of transcriptional regulators. Cytoplasmic lncRNAs can act as sponges for other transcripts and proteins, or regulate the degradation and translation of mRNAs. Moreover, lncRNAs can act as a scaffold to recruit factors that modify chromatin structure via DNA methylation and histone modifications (Beermann et al., 2016). LncRNAs occupy a large part of the genome of complex organisms. Many lncRNAs associated with chromatin modification complexes are transcribed from enhancers and nuclear phase separations of nuclear condensates and domains, suggesting that lncRNA expression is closely related to the spatial regulation of gene expression during development (Mattick et al., 2023). The mechanisms of lncRNAs enable their widespread involvement in various biological processes within cells, which are implicated in a broad range of diseases (Chen et al., 2021). Under pathological conditions, lncRNAs can contribute to the occurrence and development of cancer, osteoarthritis, diabetic kidney disease (DKD) and other diseases (Schmitz et al., 2016). Their presence has been observed in nearly all types of joint tissues and biological fluids across different species, underscoring their significance in biological systems (Ali et al., 2021). In the complex metabolic processes of articular cartilage, lncRNAs play a role in the survival of chondrocytes and the regulation of osteoarthritis-related ECM remodeling. LncRNAs can regulate chondrocyte proliferation, apoptosis, inflammation, as well as differentiation, and can also be secreted outside of the cells to participate in intercellular communication (Fujii et al., 2022). Furthermore, the remarkable stability and relative abundance of lncRNAs in circulation surpasses that of most other potential biomarkers, making them highly dependable as diagnostic targets (Badowski et al., 2022).

## 4 Regulation of the ECM by lncRNAs in osteoarthritis

### 4.1 LncRNA expression profile in osteoarthritic tissues

As key regulators of genes, abnormal expression of lncRNAs can significantly affect the balance of anabolism and catabolism of articular cartilage, and thus contribute to the occurrence and development of arthritis. A study compared lncRNA expression in lateral knee tissues of patients with mild and severe OA, and found that compared with the mild group, 52 lncRNAs were up- and 282 were significantly downregulated in knee joints of the severe group. Among them, differentially expressed lncRNAs SNHG5, ZFAS1, GAS5, and DANCR have previously been reported to promote chondrocyte proliferation (Xiao et al., 2019a). Tuerlings et al. identified 21 differentially expressed lncRNAs between preserved and lesioned OA subchondral bone of the hip and knee. Among them, AC005165.1 is also expressed in articular cartilage and tends to be expressed in subchondral bone. Therefore, AC005165.1 may be considered a potential therapeutic target for the treatment of OA (Tuerlings et al., 2022). Another research group found that 45,554 LncRNAs were differentially expressed in normal cartilage and OA tissue, among which LINC02203 (LOC727924) had the highest expression level in OA cartilage. Furthermore, LOC727924 knockdown rescued apoptosis decreased cartilage anabolism, and enhanced the catabolism of OA chondrocytes (Li et al., 2023).

The differential expression of long intergenic non-coding RNAs (lincRNAs) was also examined in the hip and knee joints affected by OA. The authors identified a total of 198 differentially expressed lincRNAs (89 up- and 109 downregulated) in the hip cartilage of OA patients compared to normal tissue. ART1, LINC01139, and NORAD were the most significantly upregulated, while LUCAT1, MEG3, and LINC01679 were the most significantly downregulated. In the knee cartilage of OA patients, 20 lincRNAs were upregulated (CRNDE, MIR22HG, and LINC01614 the most significantly), while 73 lincRNAs were downregulated (MEG3, ILF3-AS1, and LINC01089 the most significantly) (Ajekigbe et al., 2019). Therefore, selective expression of lncRNAs in different cartilage tissues, as well as differential expression in normal and pathological states, plays a role in cartilage homeostasis.

In addition to being a target for OA treatment, specifically differentially expressed lncRNAs in blood have a consistent pattern of differential expression with OA tissues, which can be used as a novel biomarker for clinical OA diagnosis. Dang et al. determined the lncRNA profiles of 98 OA patients and 76 healthy controls. It was found that the expression of long non-coding RNAs activated by transforming growth factor-β (lncRNA-ATB) was significantly downregulated in the serum of OA patients compared with the healthy control group. Furthermore, ROC curve analysis suggested that serum lncRNA-ATB might be used as a diagnostic biomarker for osteoarthritis, which can effectively distinguish OA patients from healthy controls (Dang et al., 2018). LncRNAs can also be used to investigate the pathogenesis of OA. Eight lncRNAs (AC124045.1, AL391244.1, AC106038.1, LINC00473 AL357140.2, SNHG16, AF111167.2, and AC116667.1) were found to distinguish between mild and severe pain in OA patients, and they presented a co-expression pattern in knee samples affected by different degrees of OA. These lncRNAs can therefore be used as diagnostic markers for the early onset of OA as well as to monitor the response to therapy (Chen et al., 2019a).

### 4.2 LncRNAs regulate cell proliferation, apoptosis, and ECM degradation

In OA, inflammatory factors induce cell proliferation and apoptosis, which have important implications for the integrity of chondrocytes and the ECM. The influence of lncRNAs on cell survival through the ceRNA network is directly related to the synthesis and degradation of the ECM.

During IL-1β-induced apoptosis and senescence of OA chondrocytes, knockdown of LINC00623 was found to further enhance apoptosis and increase the protein levels of cleaved-Caspase 3/7 in chondrocytes. In addition, the expression of ECM-associated type II collagen was decreased and the expression of MMP13 was increased (Lü et al., 2020). Knockdown of MCM3AP antisense RNA 1 (MCM3AP-AS) increased the level of Bcl-2 in IL-1β-induced chondrocytes, decreased the level of the apoptosis-related genes Bax and cleaved-Caspase 3 while increasing the expression of the autophagy-related genes LC3B I/II and Beclin1. In addition, lower expression of MCM3AP-AS1 was found to promote the synthesis of type II collagen and aggrecan, inhibit MMP13 expression, and protect the ECM from degradation (Xu et al., 2022).

In OA cartilage and IL-1β-induced chondrocytes, the lncRNAs MYC-Induced long non-coding RNA (MINCR) and BMPR2 were downregulated while miR-146a-5p was upregulated compared with the control group. Upregulation of MINCR can promote cell proliferation while inhibiting apoptosis and ECM degradation. It was also confirmed that MINCR can bind to miR-146a-5p, which targets BMPR2 (Li et al., 2022). The suppression of MIR22HG accelerated cell proliferation and inhibited IL-1β-induced apoptosis. ECM degradation in chondrocytes was also repressed because MIR22HG inhibition downregulated the expression of ADAMTS5 and MMP13 while increasing the expression levels of type II collagen and aggrecan. Mechanistically, MIR22HG can act as a sponge of miR-9-3p to promote the expression of ADAMTS5 (Long et al., 2021). Overexpression of lncRNA ZFAS1 also enhanced cell viability and reduced apoptosis in human chondrocyte cell line CHON-001 via the miR-7-5p/FLRT2 pathway. In addition, IL-1β stimulation upregulated the expression of MMP13 and ADAMTS5 while decreasing the levels of type II collagen and aggrecan in chondrocytes, and these expression changes were abrogated by ZFAS1 overexpression (Han et al., 2023). It has been demonstrated that lncRNA XIST is upregulated in OA cartilage tissue and IL-1β-treated chondrocytes, while apoptosis and ECM expression of chondrocytes are regulated via the miR-149-5p/DNMT3A axis. In IL-1β-treated chondrocytes, XIST knockdown promoted cell viability while inhibiting apoptosis and ECM protein degradation. Thus, the knockdown of XIST can inhibit the development of OA via the miR-149-5p/DNMT3A axis (Liu et al., 2020). Knockdown of lncRNA MALAT1 can augment the IL-1β-induced inhibition of chondrocyte proliferation and promotion of apoptosis, increase the expression of MMP-13 and ADAMTS-5, as well as decrease the expression of type II collagen and aggrecan. MiR-150-5p acts as a sponge of MALAT1 and regulates AKT3 expression. Upregulation of MALAT1 or AKT3 can enhance cell proliferation as well as inhibit apoptosis and ECM degradation, while miR-150-5p deletion salvaged the effects of MALAT1 downregulation or AKT3 deletion on IL-1β-stimulated chondrocytes (Zhang et al., 2019).

LncRNA HOX transcript antisense RNA (HOTAIR) plays a key role in the regulation of cartilage degradation and cell death. In IL-1β-induced mouse primary chondrocytes, overexpression of HOTAIR significantly promoted apoptosis and ECM degradation. Moreover, HOTAIR/miR-20b/PTEN forms a ceRNA regulatory complex. Knockdown of HOTAIR can ameliorate the cartilage injury of OA mice, promote the expression of type II collagen and proteoglycan in cartilage tissue, as well as inhibit the expression of matrix metalloproteinase MMP13 and ADAMTS-5 (Chen et al., 2020a). Lu et al. also found that overexpression of lncRNA HOTAIR increased the apoptotic rate and significantly inhibited the proliferation of chondrocytes. Moreover, HOTAIR promoted the protein expression of MMP-13 and MMP-9 but lowered the levels of type II collagen and aggrecan through the miR-107/CXCL12 pathway (Lu et al., 2021). In addition, HOTAIR was found to affect the inflammatory response and oxidative stress of chondrocytes via the miR-222-3p/ADAM10 axis. Knockout of HOTAIR was found to inhibit the expression of IL-6 and TNF-α, thereby promoting the expression of IL-10, ECM type II collagen, type X collagen, and SOX9 (Wang et al., 2021) (Table 1).

**TABLE 1:** Role of lncRNAs in chondrocyte proliferation, apoptosis, and ECM dynamics.

LncRNA	Effect on ECM	Function	Mechanism	Ref
LINC00623	Type II collagen (↑), MMP13(↓)	Chondrocyte apoptosis (−)	miR-101/HRAS ceRNA network	Lü et al. (2020)
MCM3AP-AS1	Type II collagen, aggrecan (↓); MMP13 (↑)	Chondrocyte proliferation (−), chondrocyte apoptosis (+)	miR-149-5p/Notch1ceRNA network	Xu et al. (2022)
MINCR	Type II collagen, aggrecan, SOX9 (↑); ADAMTS4, MMP3, MMP13 (↓)	Chondrocyte proliferation (+), chondrocyte apoptosis (−)	miR-146a-5p/BMPR2-down ceRNA network	Li et al. (2022)
MIR22HG	Type II collagen, aggrecan (↓); ADAMTS5, MMP13 (↑)	Chondrocyte proliferation (−), chondrocyte apoptosis (+)	miR-9-3p/ADAMTS5 ceRNA network	Long et al. (2021)
ZFAS1	Type II collagen, aggrecan (↑); MMP13, ADAMTS5 (↓)	Chondrocyte apoptosis (−)	miR-7-5p/FLRT2 ceRNA network	Han et al. (2023)
XIST	Collagen, aggrecan (↓)	Chondrocyte proliferation (−), chondrocyte apoptosis (+)	miR-149-5p/DNMT3A ceRNA network	Liu et al. (2020)
MALAT1	Type II collagen, aggrecan (↑); MMP13, ADAMTS5 (↓)	Chondrocyte proliferation (+), chondrocyte apoptosis (−)	miR-150-5p/AKT3 ceRNA network	Zhang et al. (2019)
HOTAIR	Type II collagen, proteoglycan (↓); MMP13, ADAMTS5 (↑)	Chondrocyte apoptosis (+)	miR-20b/PTEN ceRNA network	Chen et al. (2020a), Lu et al. (2021), Wang et al. (2021)
	Type II collagen, aggrecan (↓); MMP13, MMP9 (↑)	Chondrocyte proliferation (−)	miR-107/CXCL12 ceRNA network	
	Type II collagen, type X collagen, SOX9 (↓); MMP13 (↑)	Chondrocyte proliferation (−)	miR-222-3p/ADAM10 ceRNA network	

### 4.3 LncRNAs regulate chondrogenic differentiation and ECM dynamics

Chondrogenic differentiation plays a key role in the successful regeneration of cartilage because the mesenchymal stem cell-derived differentiated chondrocytes mediate stable cartilage formation. Therefore, the chondrogenic differentiation and ECM expression regulated by lncRNAs play an important role in the pathogenesis of OA.

The expression of lncRNA XIST is significantly decreased during the chondrogenic differentiation of synovium-derived mesenchymal stem cells (SMSCs) isolated from the human temporomandibular joint. Downregulation of XIST was found to promote chondrogenic differentiation, accompanied by increased expression of SOX9, type II collagen, and aggrecan, as well as enhanced proteoglycan deposition. Further studies showed that XIST could sponge miR-27b-3p to regulate the expression of ADAMTS-5 while silencing the latter restored the reduction of proteoglycan deposition caused by XIST knockdown (Zhu et al., 2021). During cartilage differentiation of bone marrow mesenchymal stem cells (BMSCs), the expression of the lncRNA colorectal neoplasia differentially expressed gene (CRNDE) and SIRT1 was increased. LncRNA CRNDE can bind to SIRT1, and its knockdown can reduce SIRT1 stability by increasing the ubiquitination level, which is mediated by E3 ligase SMURF2. Moreover, overexpression of lncRNA CRNDE increased the binding of SOX9 to the promoter of type II collagen to regulate its expression, and small interfering RNA targeting SIRT1 could reverse this binding (Shi et al., 2021). LncRNA GRASLND is upregulated during the chondrogenic differentiation of MSCs. As a downstream actor of SOX9, the silencing of GRASLND reduces the accumulation of chondroid glycosaminoglycans and type II collagen (Huynh et al., 2020). Zhu et al. found that HOTAIRM1-1 was downregulated and miR-125b was upregulated in OA cartilage, while the opposite trend was observed during chondrogenic differentiation of MSCs. In addition, HOTAIRM1-1 knockdown inhibited cartilage differentiation of MSCs. As a HOTAIRM1-1 sponge, miR-125b regulates cell differentiation by targeting bone morphogenetic protein receptor 2 (BMPR2), thus reversing the inhibitory effects of HOTAIRM1-1 on the expression of type II collagen, aggrecan, and type X collagen (Xiao et al., 2019b).

Enhancer of zeste homolog 2 (EZH2), a well-known protein with a SET domain (Hao et al., 2021), is the key catalytic component of Polycomb repressive complex 2 (PRC2), which catalyzes the tri-methylation of lysine 27 on histone 3 (H3K27me3), a well-known marker of transcriptional repression (Li et al., 2020). It has been demonstrated that LncRNA MEG3 inhibits chondrogenic differentiation of SMSCs via binding EZH2 and further regulating TRIB2. MEG3 can interact with EZH2 to induce H3K27me3 at the TRIB2 promoter, thereby inhibiting the expression of type II collagen and aggrecan (You et al., 2019). In human umbilical-cord-derived mesenchymal stem cells (hUC-MSCs), knockdown of lncRNA CIR or overexpression of ATOH8 was found to promote chondrogenic differentiation by upregulating the expression of chondrogenic transcription factor SOX9, aggrecan, and type II collagen, inducing the deposition of proteoglycan. LncRNA CIR can bind to EZH2 and inhibit ATOH8 expression through EZH2-mediated H3K27me3, thus participating in cartilage differentiation and ECM regulation (Liu et al., 2021).

In addition to directly regulating chondrocyte differentiation, lncRNAs can also indirectly mediate the regulation of chondrocyte differentiation through exosomes. As a prominent class of extracellular vesicles, exosomes serve as mediators of intercellular exchanges of signals and materials, playing an active role in numerous physiological and pathological processes (Zhang et al., 2022b). In macrophages, IL-4 or IL-13 can induce the expression of lncRNA MM2P, which in turn promotes SHP2-mediated STAT3 phosphorylation, after which p-STAT3 increases the expression of SOX9. Consequently, monocytes release exosomes rich in SOX9 mRNA and protein, promoting the differentiation of co-cultured mouse primary chondrocytes, which respond by up-regulating the expression and secretion of type II collagen and aggrecan (Bai et al., 2020). Exosome-based lncRNA therapy holds significant potential in the modulation of inflammation and the promotion of cartilage matrix reconstruction for the treatment of OA (Table 2).

**TABLE 2:** Roles of lncRNAs in chondrogenic differentiation and ECM dynamics.

LncRNA	Effect on ECM	Function	Mechanism	Ref
XIST	Aggrecan, type II Collagen, SOX9 (↓); ADAMTS5 (↑)	Chondrogenic differentiation (−)	miR-27b-3p/ADAMTS5 ceRNA network	Zhu et al. (2021)
CRNDE	Type II collagen (↑)	Chondrogenic differentiation (+)	SIRT1/SOX9 ceRNA network	Shi et al. (2021)
GRASLND	Glycosaminoglycans, type II collagen (↑)	Chondrogenic differentiation (+)	Suppressing the interferon type II signaling pathway	Huynh et al. (2020)
HOTAIRM1-1	Type II collagen, aggrecan, type X collagen, SOX9 (↓)	Chondrogenic differentiation (−)	miR-125b/BMPR2 ceRNA network	Xiao et al. (2019b)
MEG3	Type II collagen, aggrecan (↓)	Chondrogenic differentiation (−)	Inhibiting TRIB2 via methyltransferase EZH2	You et al. (2019)
CIR	Type II collagen, aggrecan (↓)	Chondrogenic differentiation (−)	Suppressing ATOH8 via methyltransferase EZH2	Liu et al. (2021)
MM2P	Type II collagen, aggrecan, SOX9 (↑)	Chondrogenic differentiation (+)	Promoting SHP2-mediated STAT3 phosphorylation	Bai et al. (2020)

### 4.4 LncRNAs directly regulate ECM-related genes through epigenetic inheritance

In addition to acting as a sponge or ceRNA for miRNAs, lncRNAs can also directly influence ECM dynamics via epigenetic regulation, such as the methylation of genes encoding matrix metalloproteases. It was demonstrated that lncRNA XIST was upregulated in cartilage tissue affected by OA, which was accompanied by downregulation of TIMP-3. In the nucleus of cartilage cells, lncRNA XIST can bind to the promoter of TIMP-3 and enhance its methylation level to reduce target gene expression (Chen et al., 2019b). TIMP-3 is an endogenous protease inhibitor, which can inhibit both MMPs and aggrecanases to effectively ameliorate the disease course of OA (Bhattacharjee and Katti, 2022). LncRNA XIST therefore promotes collagen degradation in OA chondrocytes by inhibiting TIMP-3 expression.

LncRNA WDR11 divergent transcript (lncRNA WDR11-AS1) is downregulated in OA cartilage. Moreover, knocking down lncRNA WDR11-AS1 can promote ECM synthesis (including type II collagen and aggrecan) while inhibiting the expression of metalloproteinases (including MMP3 and MMP13) in OA chondrocytes. PABPC1 is an abundant conserved eukaryotic RNA binding protein that can specifically recognize and bind poly(A)/(A)-rich sequences to regulate RNA synthesis, alternative splicing, stability, and other related processes. It was found that lncRNA WDR11-AS1 interacts with polyadenylate-binding protein cytoplasmic 1 (PABPC1), which in turn binds the mRNA of the ECM regulator SOX9 to reduce its stability, thus promoting the progression of OA (Huang et al., 2023). As a transcriptional activator, activating transcription factor 3 (ATF3) is highly selective of the AP-1 motif of MMP13 and promotes its expression. Li et al. found that RP11-364P22.2 can physically bind ATF3, and the interaction between the two plays a crucial role in the induced degradation of structural proteins of chondrocytes such as type II collagen and aggrecan. In addition, it also induces the synthesis of MMP13, which can affect cell growth and apoptosis (Li et al., 2021). In summary, LncRNAs can directly regulate the expression or control the activation of proteins related to the ECM, which are relevant to the pathological processes of OA (Table 3).

**TABLE 3:** Role of lncRNAs in directly regulating ECM-related genes through epigenetic inheritance.

LncRNA	Effect on ECM	Mechanism	Ref
XIST	MMPs, aggrecanases (↓)	Raising methylation of TIMP-3 promoter	Chen et al. (2019b)
WDR11-AS1	Type II collagen, aggrecan (↓); MMP3, MMP13 (↑)	Interacting with PABPC1 to stabilize SOX9 expression	Huang et al. (2023)
PR11-364P22.2	Type II collagen (↑); aggrecan, MMP13 (↓)	Interacting with PR11-364P22.2/ATF3 protein	Li et al. (2021)

### 4.5 LncRNAs can be used for OA therapy by regulating the ECM *in vivo*

In addition to studying the regulatory effects of lncRNAs on chondrocyte proliferation, apoptosis, and differentiation *in vitro*, many researchers are also trying to use lncRNAs as nucleic acid drugs to treat OA *in vivo*.

By injecting MEG3 overexpression plasmids into the knee joint of OA rats, it was found that MEG3 could effectively ameliorate the iatrogenic injury of cartilage, protect against cartilage degradation, and minimize the loss of proteoglycan after 8 weeks. At the same time, MEG3 was found to promote the expression of type II collagen and aggrecan while inhibiting the expression of MMP13 and ADAMTS-5 (Wang et al., 2019). LncRNA-HOTAIR, miR-17-5p, and fucosyltransferase 2 (FUT2) form a ceRNA regulatory complex during OA progression. HOTAIR and FUT2 aggravate ECM degradation and chondrocyte apoptosis, while miR-17-5p can reverse this effect. When 10-week-old SD rats were subjected to medial collateral ligament transection and destabilized medial meniscus (DMM) surgery to induce the OA model, the cartilage was thinned by intraarticular injection of HOTAIR or overexpression of FUT2. Conversely, miR-17-5p agomiR had a better protective effect on the surface layer of cartilage and surface erosion was reduced (Hu et al., 2018). Adenovirus-mediated expression of PILA Long non-coding RNA PILA stimulated NF-κB signaling in OA, induing spontaneous cartilage degradation by regulating MMP3, MMP13, and ADAMTS4 in knee joints of DMM-induced mice, thus leading to the degradation of articular cartilage and exacerbating inflammation in osteoarthritis (Tang et al., 2022b). Mao et al. reported that miR-455-3p expression was decreased while lncRNA HOTTIP and CCL3 were significantly upregulated in OA cartilage tissue and chondrocytes. HOTTIP was found to negatively regulate miR-455-3p and increase CCL3 levels in human primary chondrocytes *in vitro*. In a collagenase VII-induced mouse model of OA, it was found that cartilage degradation was significantly higher in the HOTTIP overexpression group than in the normal group and the OA group, which was accompanied by increased MMP13 expression as well as decreased type II collagen and aggrecan expression. In addition, treatment with miR-455-3p rescued the cartilage destruction caused by HOTTIP overexpression (Mao et al., 2019).

Small nucleolar RNA host gene 15 (SNHG15) plays an important role in the regulation of osteoarthritis and may be a potential target for OA therapy. Zhang et al. discovered that the overexpression of SNHG15 can promote BCL2L13 expression and mitigate cartilage degeneration in OA by sponging miR-141-3p. Compared with the control group, the expression of Caspase 3, MMP13, and ADAMTS5 was decreased. In contrast, the expression of type III collagen and aggrecan was increased in the ACLT-operated rats following intra-articular injection with a lentiviral vector expressing SNHG15. Therefore, it was demonstrated that upregulation of SNHG15 could effectively inhibit cartilage destruction and prevent the loss of articular chondrocytes (Zhang et al., 2020). SNHG15 can also regulate KLF4 expression by sponging miR-7. Overexpression of SHNG15 promoted chondrocyte formation and significantly inhibited ECM degradation in DMM-induced OA, accompanied by increased protein expression of type II collagen and proteoglycan (Chen et al., 2020b).

Developmental dysplasia of the hip (DDH) is a common hip disorder that can eventually lead to OA. In a rat model of DDH, pUbi-H19 injection without carrier effectively alleviated cartilage degeneration after 8 weeks, which was accompanied by reduced expression of MMP-3 and MMP-13 in postoperative cartilage tissue. LncRNA H19 regulates Dusp5 expression by sponging miR-483-5p, and Dups5 was found to further inhibit cartilage degradation in an ICMS-induced mouse model by activating the Erk and p38 pathways (Wang et al., 2020b). In diabetic OA model mice, intra-articular injection of ad-siRNA-PVT1 prevented ECM degradation. Adenovirus-mediated expression of siRNA-PVT1 effectively increased the expression of miR-146a and type II collagen. Moreover, joint inflammation in diabetic OA mice was reduced by inhibiting the TGF-β/SMAD4 pathway (Wang et al., 2021). Due to the important role of lncRNAs in ECM regulation, they have become potential targets for treating OA patients. These *in vivo* studies provide a crucial theoretical basis for the formulation of novel OA treatment strategies (Table 4).

**TABLE 4:** Role of lncRNAs in OA treatment by regulating ECM dynamics.

LncRNA	Effect on ECM	Function	Mechanism	Ref
MEG3	Type II collagen, aggrecan (↑); MMP13, ADAMTS5 (↓)	OA treatment (+)	miR-361-5p/FOXO1 ceRNA network	Wang et al. (2019)
HOTAIR	Type II collagen, aggrecan (↓); MMP13, ADAMTS5 (↑)	OA treatment (−)	miR-17-5p/FUT2 ceRNA network	Hu et al. (2018)
PILA	MMP3, MMP13, ADAMTS4 (↑)	OA treatment (−)	Stimulating PRMT1 and promoting NF-κB signaling	Tang et al. (2022b)
HOTTIP	Type II collagen, aggrecan (↓); MMP13 (↑)	OA treatment (−)	miR-455-3p/CCL3 ceRNA network	Mao et al. (2019)
SNHG15	Type III collagen, aggrecan (↑); MMP13, ADAMTS5 (↓)	OA treatment (+)	miR-141-3p/BCL2L13 ceRNA network	Zhang et al. (2020), Chen et al. (2020b)
	Type II collagen, proteoglycan (↑)		miR-7/KLF4 ceRNA network	
H19	MMP3, MMP13 (↓)	OA treatment (+)	miR-483-5p/Dusp5 ceRNA network	Wang et al. (2020b)
PVT1	Type II collagen (↓)	OA treatment (−)	miR 146a/SMAD4 ceRNA network	Wang et al. (2021)

## 5 Conclusion

LncRNAs have received increasing attention due to their involvement in the occurrence and development of various diseases, including cancer, as well as cardiovascular and inflammatory diseases. Recent research supporting the critical role of lncRNAs in joint and bone-related diseases may lead to promising new therapeutic strategies based on nucleic acid drugs. There is increasing evidence that many lncRNAs act as key regulators of cartilage formation and homeostasis, thereby influencing the development of OA. At present, the treatment of OA is aimed at relieving clinical symptoms, but it is difficult to block or reverse the pathological progression of OA. LncRNAs can affect the degeneration of articular cartilage by regulating the function of chondrocytes, the metabolism of the cartilage matrix, subchondral bone, and synovial cells, which is a key point in the study of new treatments for OA. The ECM is mainly distributed on the cell surface or between cells and can maintain cartilage homeostasis by forming complex network structures. In addition to structural support, there are also a large number of signal molecules in the ECM involved in intercellular signal transduction, and it is also the site of cell adhesion, proliferation, differentiation, and other cellular behaviors. In addition, as an excellent natural biomaterial, the ECM retains certain biomechanical properties and biological functions, and their advantages such as non-tissue specificity and low immunogenicity make it a good material for bone and cartilage tissue engineering for regenerative medicine.

Although OA is a multifactorial disease, lncRNAs have been shown to profoundly impact the development and progression of OA by regulating the ECM and cellular functioning of chondrocytes. The fact that altered lncRNA expression is identified at different stages of OA progression suggests that lncRNAs can be developed as biomarkers and therapeutic targets. In addition, increasing numbers of lncRNAs have been identified as participating in cartilage proliferation, apoptosis, differentiation, intercellular signaling, and regulation of key cartilage functions, which provides hope for establishing potential OA treatment strategies.
